# Differential contributions of peripheral and central mechanisms to pain in a rodent model of osteoarthritis

**DOI:** 10.1038/s41598-018-25581-8

**Published:** 2018-05-08

**Authors:** Adrian R. Haywood, Gareth J. Hathway, Victoria Chapman

**Affiliations:** Arthritis National Pain Centre, School of Life Sciences, University of Nottingham, Nottingham, NG7 2UH United Kingdom

## Abstract

The mechanisms underlying the transition from acute nociceptive pain to centrally maintained chronic pain are not clear. We have studied the contributions of the peripheral and central nervous systems during the development of osteoarthritis (OA) pain. Male Sprague-Dawley rats received unilateral intra-articular injections of monosodium iodoacetate (MIA 1 mg) or saline, and weight-bearing (WB) asymmetry and distal allodynia measured. Subgroups of rats received intra-articular injections of, QX-314 (membrane impermeable local anaesthetic) + capsaicin, QX-314, capsaicin or vehicle on days 7, 14 or 28 post-MIA and WB and PWT remeasured. On days 7&14 post-MIA, but not day 28, QX-314 + capsaicin signficantly attenuated changes in WB induced by MIA, illustrating a crucial role for TRPV1 expressing nociceptors in early OA pain. The role of top-down control of spinal excitability was investigated. The mu-opioid receptor agonist DAMGO was microinjected into the rostroventral medulla, to activate endogenous pain modulatory systems, in MIA and control rats and reflex excitability measured using electromyography. DAMGO (3 ng) had a significantly larger inhibitory effect in MIA treated rats than in controls. These data show distinct temporal contribtuions of TRPV1 expressing nociceptors and opioidergic pain control systems at later timepoints.

## Introduction

Osteoarthritis (OA) is a common form of degenerative joint disease and a major cause of joint pain worldwide^1^. Structural changes within the joint are well characterised, however the mechanisms of OA pain are less clearly defined^2,3^. Patients with OA pain have symptoms consistent with hyperalgesia including altered lower limb reflexes^4^, lowered mechanical pain thresholds at sites distal to the diseased joint and facilitated temporal summation^5^. Around 30% of patients exhibit features consistent with central sensitisation mechanisms^6^. Synovitis is significantly associated with OA pain^7,8^ and increased levels of pro-inflammatory mediators in synovial fluid sensitise nociceptors^9^. These nociceptors are primary afferent sensory neurons which innervate multiple components of the joint and detect changes in the local environment, as well as being potential sources of nociceptive factors themselves. Intra-articular injection of the local anaesthetic lidocaine significantly reduced pain scores in OA patients^10^, supporting the importance of a peripheral drive to OA pain, however whether all sensory fibres, or just subsets, are sensitised in OA is unknown.

As well as primary afferent sensory input to the spinal cord and higher brain centres, supraspinal networks powerfully control spinal excitability and hyperalgesia. Preclinical studies support the contribution of supra-spinal mechanisms to the maintenance of chronic pain^11^ including in models of OA pain^12^. Descending control mechanisms arise from multiple supra-spinal sites, changes in neuronal activity in the rostral ventromedial medulla (RVM)^11^ has a fundamental role in the modulating spinal dorsal horn excitability^13^. Descending modulation is altered in OA patients^14,15^, however identifying the underlying structures and mechanisms responsible and the timecourse over which central mechanisms become clinically significant necessitates the use of translationally relevant animal models.

Although a range of OA pain models have been developed, given the complex nature of OA in humans there is no single ‘gold standard’ model which can replicate all aspects of human OA^16^. Recent reviews confirm the value of both surgical and chemical models^17^. The mono-sodium iodoacetate (MIA) model of OA has been used extensively to study structural features of OA^18^ and pain behaviour^19,20^ in the rodent. Intra-articuar injection of MIA results in extensive cartilage degradation and subchondral bone changes at later timepoints^19^, which is dose and time dependent. Studies using an intermediate dose report structural changes to the joint commensurate with that seen in human OA joints^21^. Concomitant MIA-induced pain behaviour (weight bearing asymmetry (WB) and lowered paw withdrawal thresholds (PWT)) models aspects of activity-related pain and altered distal pain pressure thresholds in OA patients^19,22^. This model is also associated with hallmarks of of spinal^20,23^ and supraspinal^24^ sensitization.

We hypothesised that there are differential contributions of the peripheral and central nervous system to MIA induced OA pain over time, which are relevant to the development of novel treatment approaches.

## Results

Joint pathology was assessed post-mortem. Macroscopic scoring in six compartments of the knee in MIA and saline injected rats showed significantly higher scores in the treated group than in the controls (Supplemental Fig. 1). Further macroscopic scoring increased significantly over time.

### Targeted inactivation of joint nociceptors attenuates pain evoked by intra-articular injection of NGF

**Figure 1:**
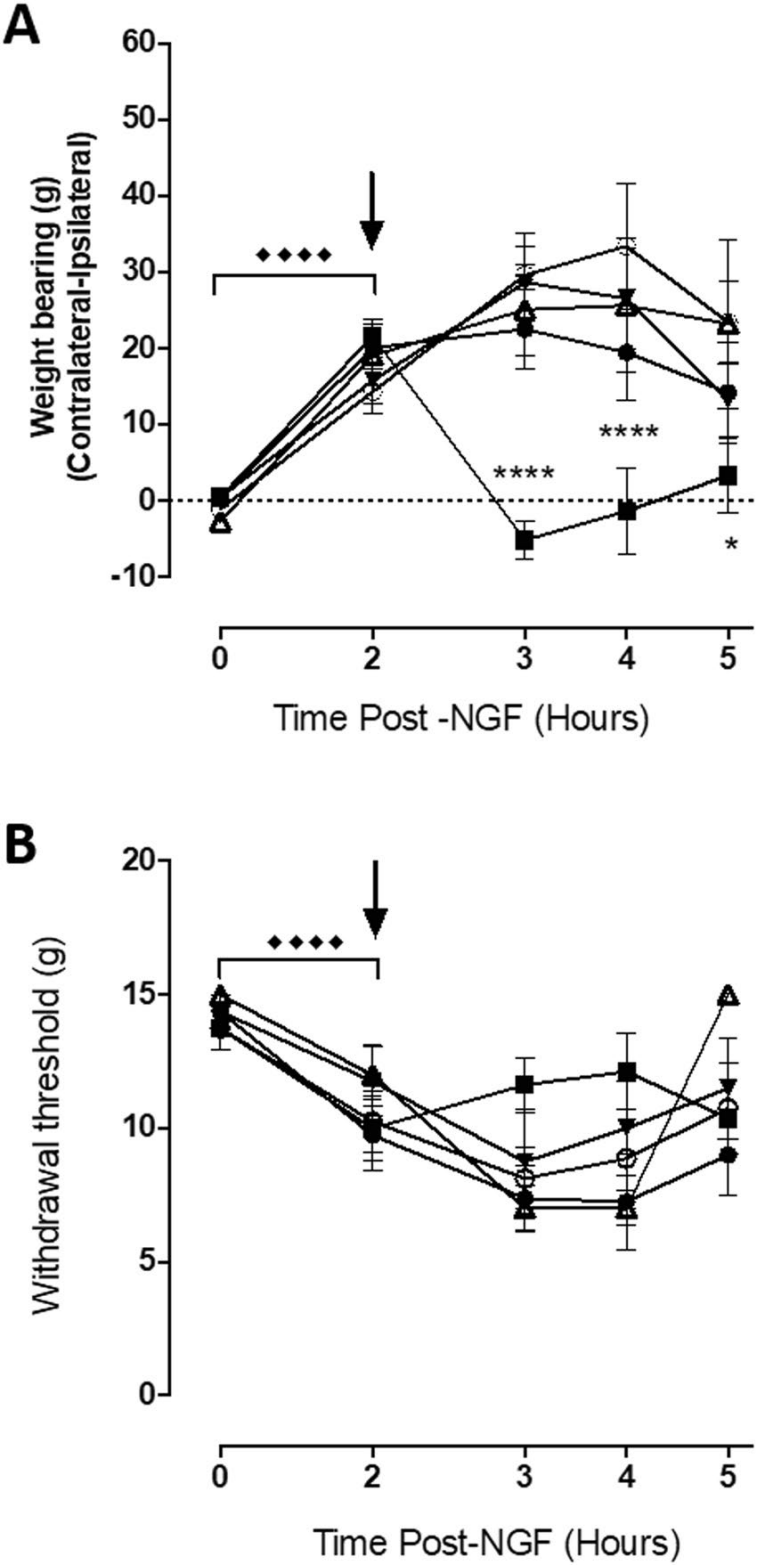
Intra-articular inactivation of TRPV1 expressing nociceptors reverses NGF induced pain. Intra-articular NGF (10 μg/50 μl) produced significant WB asymmetry (**A**) and reductions in PWTs (**B**) at 2 hours post-injection. Subsequent injection (indicated by the black arrow) of membrane impermeable local anaesthetic QX-314 alone (●) did not alter NGF-induced pain behaviour. Concomitant injection of QX-314 (1 mg/50 μl) with capsaicin (1 μg per μl) (■)significantly reversed WB asymmetry up to 2 hours post-injection (**A**), but had no effect on PWTs (**B**). ● = QX-314, ■ = QX314 + capsaicin, Δ = capsaicin, ▲ = lidocaine, ○ = vehicle. Changes in PWT or WB following NGF injection were performed using a 2-way ANOVA (treatment x time) with Dunnett’s multiple comparison test comparing WB and PWT to the measurement. ◆◆◆◆ < 0.0001 (time 0 vs 2hrs post NGF). *p < 0.05, **p < 0.01, ***p < 0.001, ****p < 0.0001 (Dunnett’s; 2hrs postNGF vs pharmacological intervention)). All data are presented as mean ± SEM.

The first study confirmed whether targetted inactivation of joint nociceptors reversed pain behaviour evoked by a well studied sensitising agent. NGF is a key mediator of OA pain and is able to sensitise nocieptors in the joint. The ability of the membrane impermeable local anaesthetic QX314 with capsaicin to block acute joint pain responses following intra-articular administration of NGF was determined. Intra-articular injection of NGF (10 ug) evoked significant WB asymmetry (p < 0.0001;Fig. 1A) and a reduction in PWTs (p < 0.001; Fig. 1B) 2 hours post injection of NGF. Concomitant intra-articular injection of QX-314 (1 mg/50 ul) with capsaicin (1 ug/ul) significantly attenuated NGF-induced WB asymmetry (p = 0.0004; Fig. 1A), compared to all other intra-articular interventions. Intra-articular injection of QX-314 (1 mg/50 ul) with capsaicin (1 ug per ul) did not however alter NGF-induced changes in PWT (p > 0.05; Fig. 1B). Intra-articular injection of QX-314 (1 mg/50 ul), lidocaine (1 mg/50 ul), capsaicin (1 ug/ul; Sigma) or vehicle (5% EtOH, 5% tween-20, 90% saline 0.9%) did not alter NGF-induced WB asymmetry or PWTs. These data show that in an established model of joint hyperalgesia, targeted inactivation of TRPV1 expressing C-fibres are able to reverse WB asymmetry, but not lowered PWTs.

### MIA-induced weight bearing asymmetry is dependent upon TRPV1 positive joint nociceptor activation at early time points

**Figure 2:**
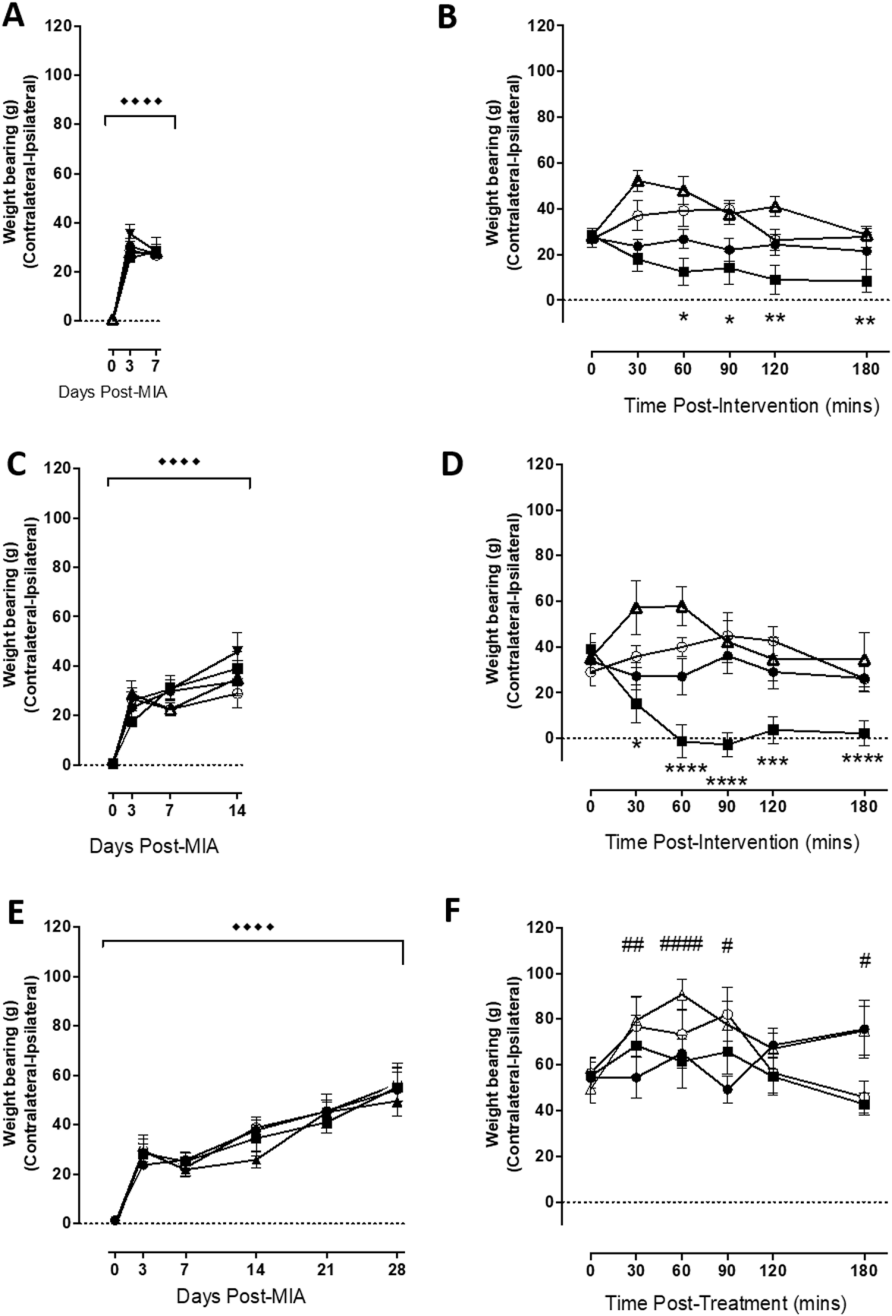
Inactivation of TRPV1 expressing nociceptors reverses MIA induced alterations in weight-bearing up to Day 14. Intra-articular injection of MIA significantly altered WB 7 (**A**), 14 (**C**) and 28 (**E**) days following administration (Dunnett’s; ^◆◆◆◆^p < 0.0001). At Days 7 (**B**) and 14 (**C**) co-administration of QX-314 and capsaicin reversed MIA induced alterations in WB asymmetry at Day 7 and 14 but not at Day 28 (**F**), compared pre-injection values. None of the other drugs significantly altered WB asymmetry at Day 7 or 14. However at Day 28, capsaicin significantly enhanced WB asymmetry compared to pre-drug, baseline values. ● = QX-314, ■ = QX314, Δ + capsaicin, = capsaicin, ○ = vehicle. Effects of interventions on WB asymmetry were performed using a two-way repeated measures ANOVA with Dunnett’s post-hoc test. *p < 0.05, ***p < 0.001, ****p < 0.0001 (* QX-314 + capsaicin vs respective baseline value). ^#^p < 0.05, ^##^p < 0.01, ^####^p < 0.0001 (# capsaicin alone vs respective baseline value). All data are presented as mean ± SEM.

Next we studied the contributions of nociceptors innervating the joint versus central pain facilitating pathways to hyperalgesia in a model of joint damage over time. Intra-articular injection of MIA (1 mg/50 ul) resulted in significant WB asymmetry at day 7 (p < 0.0001; Fig. 2A), day 14 (p < 0.0001; Fig. 2C) and day 28 (p < 0.0001; Fig. 2E) compared to baseline values. In our NGF study lidocaine failed to reverse changes in PWT or WB, whereas targeted inactivation of TRPV1 expressing nociceptors did. We therefore decided to investigate the contribution of these TRPV1 positive sensory nerve fibres in MIA induced pain behaviours.

In rats at 7 days following intra-articular injection of MIA, intra-articular injection of QX-314 (1 mg/50 ul) with capsaicin (1 ug/μl) significantly reduced WB asymmetry (Fig. 2B) compared to baseline. Intra-articular capsaicin significantly potentiated WB asymmetry 30 and 60 mins post application, compared to baseline (Fig. 2B). None of the other intra-articular manipulations had significant effects on WB in MIA treated rats at this time point compared to baseline. Comparisons of the mean response to these intra-articular manipulations between groups over the entire 180 min observation period demonstrated significant differences between QX-314 + capsaicin treated rats with those treated with just capsaicin (p < 0.001), or vehicle (p < 0.05; Table 1).

**Table 1:** The effect of intra-articular pharmacological treatments at days 7, 14 and 28 on WB asymmetry in MIA pre-treated rats. Comparisons between QX-314 + capsaicin treated rats have been made with QX-314, capsaicin, lidocaine and vehicle treated rats. Comparisons were made with a repeated measure 2-way ANVOA with Sidak’s post-test. *p < 0.05, **p < 0.01, ***p < 0.001, ****p < 0.0001.

Days Post-MIA	QX314 vs QX314 + capsaicin	Capsaicin vs QX314 + capsaicin	Lidocaine vs QX314 + capsaicin	Vehicle vs QX314 + capsaicin
7	—	****	**	*
14	*	***	****	**
28	—	—	—	—

The same intra-articular drug applications were made in a separate cohort of rats 14 days after MIA injection. At this timepoint only QX-314 (1 mg/50 ul) with capsaicin (1 ug/μl) significantly reduced WB asymmetry compared to baseline, none of the other treatments altered WB asymmetry (Fig. 2D). QX314 and capsaicin co-administration had a significantly different effect to all other drug treatment groups at day 14 post-MIA over the entire 180 min observation period (see Table 1).

Finally at Day 28 post-MIA the same pharmacological treatments were also applied intra-articularly in another cohort of rats. At this timepoint administration of QX-314 (1 mg/50 ul) with capsaicin (1 ug/μl) had no effect upon WB asymmetry compared to baseline values. Capsaicin however was able to increase WB asymmetry at this timepoint.

**Figure 3:**
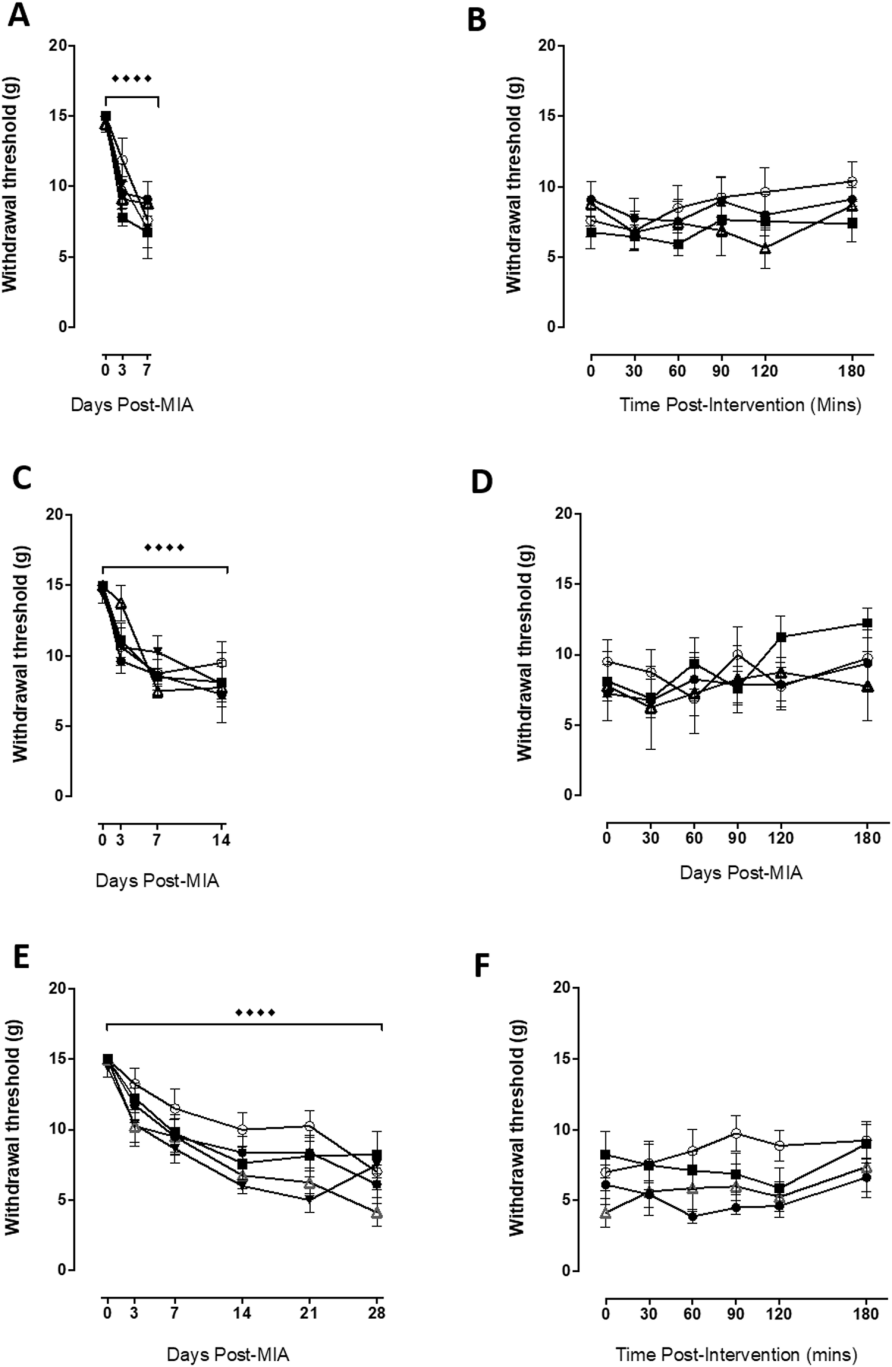
QX-314 + capsaicin is unable to effect MIA induced distal allodynia. Intra-articular injection of MIA significantly lowered PWTs 7 (**A**), 14 (**C**) and 28 (**E**) days following administration (Dunnett’s; ^◆◆◆◆^p < 0.0001). Subsequent pharmacological treatments (intra-articular injection) were without effect at any of the post-MIA timepoints. ● = QX-314, ■ = QX314, Δ + capsaicin, = capsaicin, ○ = vehicle All data are presented as mean ± SEM.

Intra-articular injection of MIA (1 mg/50 ul) resulted in significant PWT asymmetry at day 7 (p < 0.0001; Fig. 3A), day 14 (p < 0.0001; Fig. 3C) and day 28 (p < 0.0001; Fig. 3E). Unlike WB, intra-articular administration of QX-314 with capsaicin, QX314, capsaicin or vehicle at each of these timepoints had no effect upon PWT when compared to each other or their respective baseline (pre-local anaesthetic) values (Fig. 3B,D–F).

### MIA-induced central sensitization: facilitation of EMG responses

To advance understanding of the contribution of central mechanisms to MIA-induced pain over time, EMG responses were recorded at different timepoints and the effects of altering the output of the RVM on these responses were quantified in separate groups of rats. Our EMG approach permits discrete pharmacological manipulation of brainstem structures, whilst preserving spinal reflexes and has been used to investigate opioidergic signalling in descending pain control systems previously^25^. It also overcomes animal welfare concerns associated with supraspinal microinjections in freely-behaving rats. EMG activity is directly correlated with spinal excitability. Changes in spinal excitability are associated with reductions in thermal and mechanical threshold and central sensitisation^26^.

**Figure 4:**
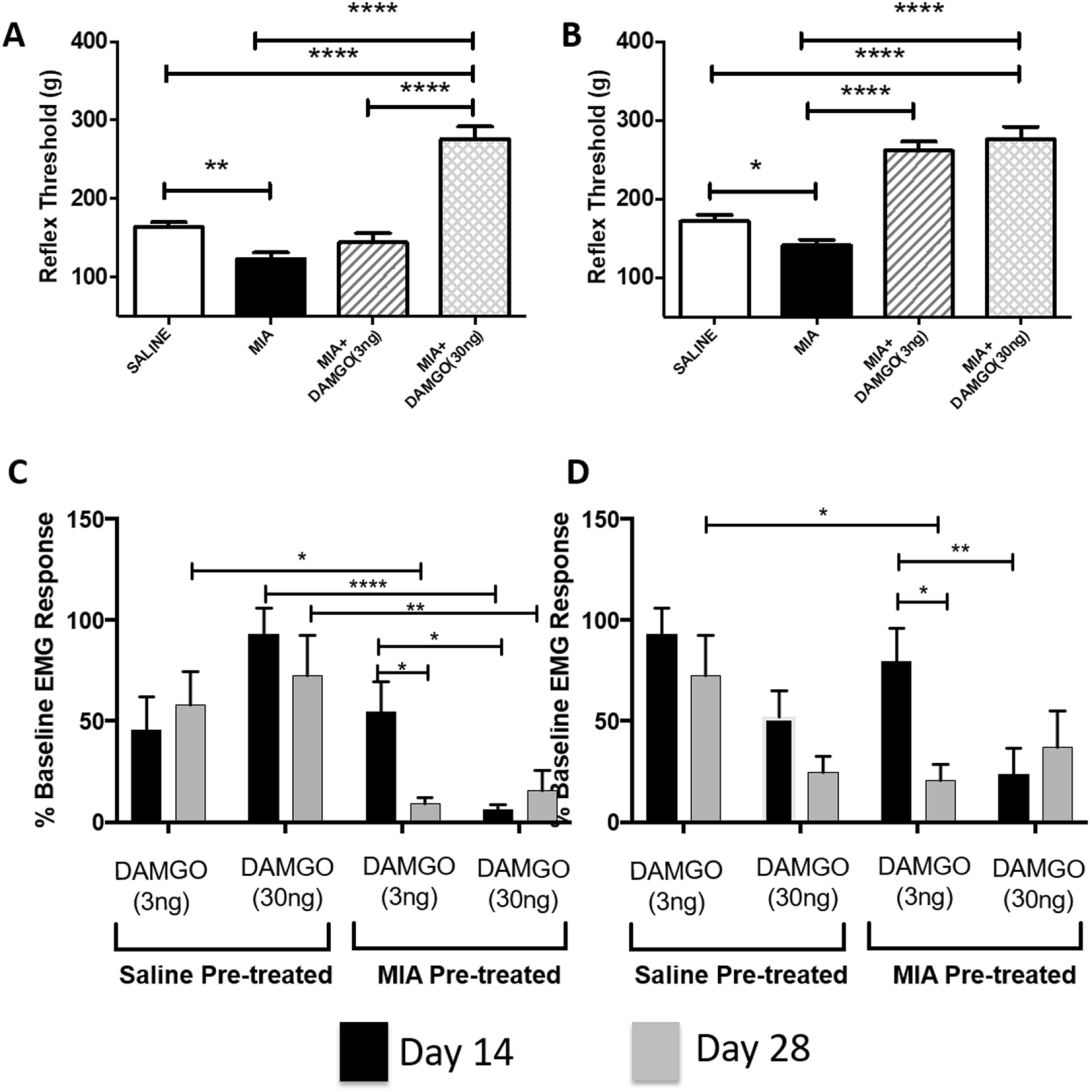
MIA leads to a increased sensitivity of endogenous opioid signaling within the RVM. EMG characterization of the nociceptive withdrawal reflexes in rats. Thresholds for mechanically evoked EMGs were significantly reduced in MIA-treated rats compared to saline rats at 14 (**A**) and 28 days (**B**) following intra-articular injection of MIA. Intra-RVM administration of 3 ng DAMGO did not alter MIA induced reductions in EMG threshold at Day 14 (**A**), but did reverse this reduction at Day 28 in MIA rats (**B**). Intra-RVM administration of DAMGO (3 ng) significantly inhibited EMG response magnitude to threshold vFh stimulation in saline (white bars) and MIA (black bars) treated rats at Day 14 compared to baseline (pre-drug) values. Low dose DAMGO (3 ng) was reduced EMG responses to threshold stimuli (**C**) in MIA treated rats at both Day 28 compared to saline treated controls but high dose DAMGO (30 ng) reduced responses at both Day14 and 28. A similar but not identical pattern was seen following DAMGO to responses to suprathreshold stimuli (**D**). Threshold determinations were compared using a one-way ANOVA with Bonferroni post-test. EMG magnitude and duration were compared between groups using a two-way repeated measures ANOVA with Sidak post-hoc test **p < 0.01, ***p < 0.001, ****p < 0.0001. Day 14: MIA (n = 27 rats); Saline (n = 29 rats). Day 28: MIA (n = 50 rats); Saline (n = 38 rats). All data are presented as mean ± SEM.

At 14 and 28 days following model induction there was a significant reduction in the threshold for mechanically-evoked EMG responses in MIA-treated rats, compared to saline controls (p = 0.0002 and p = 0.0071 respectively; Fig. 4A,B). At day 14 in MIA-treated there was no significant difference in the overall magnitude of EMG responses (EMG responses to all hairs) in MIA-treated rats, compared to saline rats (p = 0.9; Fig. 4C). However, by day 28 overall EMG responses in MIA rats were increased in magnitude (p = 0.025; Fig. 4D), compared to saline rats. Post-hoc analysis at Day 28 showed a significant difference in the mean EMG response evoked by the supra-threshold hair (p = 0.0025 Fig. 4D) in MIA rats, compared to saline rats. The duration of the EMG response was also quantified. No significant interaction was observed between groups at 14 days following intra-articular injection of MIA (p = 0.2484; Fig. 3E). At day 28 the EMG response duration was significantly longer in MIA-rats, compared to saline-treated rats (p = 0.0312; Fig. 4F). Post-hoc analysis revealed a significantly longer duration of supra-threshold evoked EMG responses in the MIA-rats, compared to saline-treated rats at this late timepoint (p = 0.003; Fig. 4F).

### Effects of intra-RVM lidocaine and DAMGO on EMG responses in the MIA model

Intra-RVM microinjection sites are were determined post-mortem and are shown in Supplemental Fig. 2. To understand the potential influence of descending control pathways in the MIA model of OA pain, we first investigated the effect of an intra-RVM injection of lidocaine on EMG responses in MIA rats. The RVM contains projections of at least two populations of neurons that are able to inhibit or facilitate pain responses at the spinal cord level. Intra-RVM lidocaine treatment had no significant effect on any parameter of the EMG responses in MIA-treated rats compared to saline controls (see Supplemental Fig. 3). Given the generalised effects of lidocaine on this mixed population of neurones in the RVM^27^, the effect of a more specific intervention using a pharmacological tool that activates endogenous pain control systems was studied. The effects of injection of the mu-opioid receptor (MOR) agonist DAMGO into the RVM of MIA- and saline-treated rats on EMG thresholds and the magnitude of responses were compared to the effects of intra-RVM injection of saline in separate groups of rats. At day 14 in MIA rats, intra-RVM DAMGO (3 ng) did not alter the threshold of mechanically-evoked EMG responses (Fig. 4A), but 30 ng DAMGO significantly increased thresholds compared to MIA rats which received intra-RVM saline as a control (p < 0.0001) and MIA rats that received the low dose of intra-RVM DAMGO (3 ng)(p < 0.0001; Fig. 4A). In the Day 28 MIA group of rats, both 3 ng and 30 mg intra-RVM DAMGO significantly increased EMG thresholds in MIA rats, compared to intra-RVM-saline in MIA rats (p < 0.0001 in both comparisons Fig. 4B). These data show altered sensitivity of the RVM to activation of opioidergic signalling at later time points in the MIA model. Intra-RVM injection sites were confirmed at the end of each recording session (Supplementary Figure 1).

To further analyse the change in opioid sensitivity we performed post-hoc analyses of responses to threshold (Fig. 4C) and suprathreshold (Fig. 4D) mechanical stimuli in MIA and saline rats at Day 14 and 28 in separate groups of rats.

#### Threshold Responses

At day 14 intra-RVM DAMGO (3 ng) had the same effect in both saline and MIA treated rats. However at Day 28 3 ng DAMGO caused significantly greater analgesia in MIA treated rats than in saline controls (p < 0.05). Administration of 30 ng intra-RVM DAMGO to MIA treated rats at both day 14 (p < 0.0001) and day 28 (p < 0.01) produced significantly more analgesia than when the same concentration was applied to saline controls. In MIA treated rats low dose intra-RVM DAMGO (3 ng) had a significantly greater analgesic effect at day 28 compared to day 14 (p < 0.05).

#### Suprathreshold Responses

Responses to these higher intensity stimuli were less complex than to threshold stimuli. Low dose DAMGO (3 ng) had the same effect in MIA and saline treated rats at Day 14 but had a significantly greater effect in MIA treated rats at Day 28. DAMGO (30 ng) had a greater effect at Day 14 than 3 ng DAMGO (p < 0.01). Low dose of DAMGO was more efficacious in producing analgesia at Day 28 than it was at Day 14 (p < 0.05).

These data illustrate that the sensitivity μ-opioid receptor activated endogenous pain control systems in the RVM are increased at later time points in the MIA model of OA pain, indicative of alterations in endogenous opioidergic signalling.

## Discussion

Here we provide new evidence for a differing contribution of peripheral inputs to the early versus late stages of MIA-induced OA pain and for an alteration in opioid mediated top-down control of spinal excitability and central sensitization in the late stage of the model. We have demonstrated that behavioural responses following the induction of acute inflammatory joint pain induced by NGF or the earlier stages of longer lasting OA-like joint pain induced by MIA were significantly attenuated by transient inactivation of TRPV1 expressing nociceptors in the affected knee using QX-314 plus capsaicin. The ability of QX-314 plus capsaicin to attenuate pain behavioural responses at early but not late stages of the MIA model suggests either a significant phenotypic switch in the peripheral input driving pain (i.e. within nociceptors), or an increased central contribution to the maintenance of this pain. Appreciating the presence and timecourse of this switch is important in targeting the pharmacological management of OA pain to the appropriate target (i.e. peripheral inflammation versus altered CNS function).

Intra-articular injection of NGF resulted is an acute WB asymmetry and lowered PWTs consistent with NGF-induced sensitisation of TRPV1 channels via activation of TrkA receptors, and facilitated responses of these nociceptors to capsaicin^28^. Acute intra-articular injection of QX-314 plus capsaicin had a selective inhibitory effect on WB asymmetry, but not lowered PWTs. In the absence of the TRPV1 ligand capsaicin, QX-314 did not alter WB asymmetry, consistent with other studies using this pharmacological intervention to target TRPV1 positive sensory nerves^29^. NGF-induced lowering of hindpaw withdrawal thresholds (distal allodynia), thought to arise from alterations in the central processing of nociceptive inputs, was unaffected by sensory specific blockade of joint nociceptors by QX-314 plus capsaicin. NGF-induced distal allodynia persists up to 3 days post-injection^30^ and the continued presence of distal allodynia following the blockade of TRPV1 expressing sensory input from the joint at 2 hours following NGF injection, suggests that relatively short term activation of sensory nerves by NGF in the joint is sufficient to induce long lasting central changes^31^ that are not reversed by the removal of peripheral input.

In the MIA model of joint pain, rats exhibited significant pain behaviour (WB asymmetry and lowered PWTs) at the earlier timepoints studied (7 and 14 days post-injection). At these two timepoints, intra-articular injection of QX-314 plus capsaicin significantly inhibited WB asymmetry, consistent with the known peripheral joint inflammation, increased spontaneous firing of nociceptive afferents at 14 days in the MIA model^32^ and that local blockade of TRPV1 in the joint reversed established MIA-induced pain behaviour^33^. Consistent with our findings in the acute NGF model, QX314 plus capsaicin treatment had no effect on lowered PWTs in MIA rats at day 7 or day 14. Overall these data support a major contribution of TRPV1 positive sensory joint fibres to the WB asymmetry, but not lowered PWTs, at the early stages of the MIA model of OA pain. Recently it has been shown that chemogenetic inhibition of NaV 1.8 expressing neurons blocks hyperalgesia in early experimental knee osteoarthritis^34^ which supports our data that TRPV1 expressing nociceptors (a subset of Nav 1.8 expressing C-fibres) underpin the development of joint pain.

The lack of efficacy of intra-articular injection of lidocaine in reversing either NGF or MIA mediated WB asymmetry is surprising. The concentration of lidocaine that was used in the studies is the same that has been used previously^35^. We consider a period of at least 30 mins is necessary after the intra-articualr injection of lidocaine (under general anaesthesia) is necessary before further behavioural assessments can be made to mitigate against anaesthetic impacting upon behaviour. Previous studies which have shown a positive effect of lidocaine upon pain behaviour in this context and the lack of efficacy in our studies of lidocaine may well reflect the fact that compared to these studies the volume used was smaller^35^. Similarly intra-RVM lidocaine failed to alter descending control in our study. Although the concentration and volume of lidocaine microinjected in our study has been used successfully by others^36^ it is at the lower end of range used in other studies^37^ and this may explain the lack of efficacy in our hands.

The data demonstrate an effect of QX-314 + capsaicin upon WB but not on VF evoked withdrawal thresholds. This would indicate that the selective inactivation of TRPV1 + ve C-fibres only effects MIA induced primary hyperalgesia, and has no effect upon the distal allodynia observed by reduced mechanical withdrawal thresholds evoked by stimulating the ipsilateral plantar footpad. C-fibres therefore drive weight bearing asymmetry but play little to no role in the maintenance of central sensitisation that are thought to underpin allodynia.

In stark contrast to day 7 and 14, intra-articular injection of QX-314 plus capsaicin did not alter WB asymmetry at 28 days in the MIA model. These data suggest that at this timepoint factors other than TRPV1 expressing sensory nerves play a greater contribution to this pain phenotype. Day 28 represents a timepoint at which all behavioural changes associated with experimental OA have become fully developed^38^. The lack of efficacy of QX-314 + capsaicin may represent the recruitment of additional/different sensory nerves, or a greater contribution of spinal/supra-spinal mechanisms to WB asymmetry. Previous work from our group has demonstrated markers of central sensitization indicative of the transition from acute to chronic pain mechanisms at this timepoint in the MIA model. Specifically, ipsilateral spinal expression of GFAP immunofluorescence is significantly elevated in this model at 28 days^20^, which is linked to hyperalgesia in other models of chronic pain. Previously MIA has been shown to result in increased afferent drive that is refractory to the anti-inflammatory actions diclofenac or the TRPV1 antagonist HC030031^39^. This work concluded that afferent drive is essential at 14 days post MIA for pain but that TRPV1 did not play a role. Our data add to this by illustrating that afferent drive from TRPV1 expressing neurons is essential in the early stages of MIA induced pain, including day14, these are not essential in later stages and that whilst the TRPV1 receptor may not be essential in MIA induced WB and PWT the neurons they are expressed on are.

It is increasingly recognised that EMG responses are sensitive to disease related alterations in CNS processing^23,40^ and have great utility for the assay of central sensitisation associated with chronic pain in both animals and humans. At 14 and 28 days in the MIA model, rats exhibited reduced reflex thresholds, and at day 28 rats had significantly enhanced reflex magnitude. These findings are consistent with a previous animal study of OA^23^ and clinical studies in OA patients^4^ reporting the presence of central sensitization using EMG as an assay of excitability. EMG reflex responses are sensitive to the inhibitory effects of analgesics which act either directly in the spinal cord, or within supraspinal centres that innervate the spinal cord^41,42^. The RVM is the major source of both descending inhibitory and facilitatory influences over DH excitability^43^. RVM neurophysiology is driven by three types of neurone: ON cells which promote chronic pain states and are the only RVM cell type to be directly inhibited by MOR agonists^44^; OFF cells which decrease their basal activity just before a noxious stimulus; NEUTRAL cells show no consistent change in their firing patterns to noxious stimulation. Only ON cells express MOR and these receptors suppress the excitability and therefore produce a direct inhibition of descending facilitation [39]. However ON and OFF cells are reciprocally connected and inactivation of ON cells will also increase activity in OFF cells therefore amplifying DAMGO mediated descending inhibition of DH excitability. In the present study low dose DAMGO (3 ng) was able to inhibit spinal excitability in both MIA and saline treated rats in response to threshold, but not suprathreshold stimuli. This effect of DAMGO in saline treated rats is unsurprising as the drug will have inhibited low level basal descending facilitation and enhanced descending inhibition. Thus the low dose of DAMGO can provide enough descending inhibitory drive to overcome the increased excitatory input from the MIA effected knee combined with the larger vFh evoked noxious input emanating from the foot. Our major finding was that a lower dose of DAMGO had significant inhibitory effects on EMG thresholds and magnitude of evoked EMG responses at 28 days in the MIA rats, but not in MIA rats at the earlier timepoints studied. The fact that the higher dose of DAMGO (30 ng) significantly inhibited EMG responses in both groups at both times whereas the lower dose was most efficacious in MIA rats at Day 28 indicates increased RVM contributions to MIA induced pain at the later timepoint and/or increased opioid sensitivity in late stage OA pain. These data extend previous indirect evidence for a contribution of supraspinal sites to MIA-induced pain provided by studies of the effect of transection of the spinal cord at a thoracic level on pain behaviour^23^ and that blockade of spinal cord 5-HT_3_ receptors reversed MIA-induced hypersensitivity of spinal dorsal horn neurones^24^. There may appear to be an incongruity between the DAMGO and QX-314 + capsacin data in as much as vFh evoked activity was significantly effected by the former by unaffected by the latter. This difference is completely logical when it is considered that QX-314 + capsacin only impacted upon sensory neurones that innervated the joint and a limited somatotopic region within the DH, whereas RVM administered DAMGO will have decreased DH facilitation and increased descending inhibition throughout the entire DH as the RVM has no somatotypic map and RVM neurons are known to respond to stimulation across the body.

Our demonstration of a significant change in the opioidergic system sensitivity in the RVM in this model of OA pain has considerable significance, both in terms of understanding the neurophysiological substrates driving OA pain and the importance of targeting central mechanisms of pain for treatment. OA patients exhibit alterations in supraspinal responses to pain as evidenced by increased activity within the cingulate cortex, thalamus and amygdala^45^. In addition, atrophy of the thalamic^46^ and cortical gray matter^47^ that can be reversed by arthroplasty. Although, changes in these centres do not directly modulate spinal pain processing they will alter activity within brain centres that do impact upon DH excitability^48^. Altered diffuse noxious inhibitory control^49^ (indicating alterations in descending inhibition) and increased activation of the periaqueductal gray^50^ (a structure that mediates its effects on DH excitability indirectly via the RVM) have both been demonstrated in OA patients. The extent to which opioidergic systems in the brain are altered in OA patients has yet to be reported. The contribution of a supraspinal drive of central sensitization, mediated by the opioidergic system, in established OA pain may reflect a functional up-regulation of opioid receptors in the RVM and/or a result of a loss of endogenous mu opioid ligand, but this needs experimental confirmation.

In conclusion we have shown that there are specific periods during the development of pain in a model of OA in which peripheral input by TRPV1 positive nociceptors innervating the affected joint are a pre-requisite for pain on loading of the joint. At later timepoints TRPV1 positive nociceptors are involved to a much lesser extent, but neuromodulatory influences from the RVM appear to play a greater role in mediating the increased excitability of the DH associated with this model of OA pain. This time-dependent role of the peripheral and central nervous system in the development and maintenance respectively of chronic pain states is in agreement with that seen in other experimental pain states^37^. Further investigation is required to elucidate how afferent sensory input leads to changes in brainstem pain modulation.

## Materials and Methods

### Animals

Male Sprague-Dawley rats (180–200 g; Charles River, UK) were used and kept on a 12 h dark/light cycle, group housed with free access to food and water. All procedures were performed in accordance with and specifically licenced under the Animals (Scientific Procedures) Act 1986 following ethical review by The University of Nottingham Animal Welfare Ethical Review Body and the UK Home Office. All studies took place in accordance with the ARRIVE guidelines. At the end of experiments, rats were killed by overdose of anaesthetic followed by cervical dislocation.

### Model induction and behavioural testing

Rats were anaesthetised with isoflurane (3% in 99% O2) and received a single injection through the infra-patellar ligament of the left knee, of one of the following: 1) recombinant rat beta-nerve growth factor [NGF; 10 ug/50 ul; R&D Systems, dose based on^30^] to model acute joint pain, or 2) mono-sodium iodoacetate (MIA; 1 mg/50 µl; Sigma U.K.)^33^ to model chronic joint pain. Pain behaviour was assessed as changes in hindlimb weight-bearing (WB) and changes in mechanical hindpaw withdrawal thresholds (PWT) as previously described^21^ by an experimenter blinded to all treatments. Baseline behavioural measurements were taken prior to intra-articular injections. In the NGF model, behaviour was tested pre and 2 hrs post injection. Following intra-articular injection of MIA, pain behaviour was assessed at day 7, 14, 21 and 28.

### Intra-articular drug treatments

Following single injection of NGF or MIA, rats recieved a second pharmacologcial intervention at the timepoints as follows: NGF-treated rats at 2 hours post model induction; MIA-treated rats at 7, 14 or 28 days post model induction. The treatment was one of the following as an intra-articular injection in the ipsilateral knee under isoflurane anaesthiesia: lidocaine (1 mg/50 ul; Sigma U.K.); QX-314 (N-Ethyllidocaine bromide, 1 mg/50 ul; AbCam); capsaicin (1 ug per ul; Sigma U.K.); QX-314 (1 mg/50 ul) with capsaicin (1 ug per ul); or vehicle (5% EtOH, 5% tween-20, 90% saline 0.9%) (see Fig. 1). Effects of treatments on WB asymmetry and PWTs were assessed at 30, 60, 90, 120, and 180 mins post treatment. The local anaesthetic QX314 is membrane impermable, however, co-administration with a low dose of the TRPV1 ligand capsaicin selectively inactivates the TRPV1 positive subset of sensory neurones^29^.

### EMG recordings and RVM Microinjections

EMG responses and the influence of modulating the output from the RVM on these responses was characterised at different stages of the model of OA pain. These studies were performed in anaesthetised rats as this permits responses to a range of sub- and suprathreshold stimuli to be assessed. Additionally this permits more accurate microinjection of pharmacological tools and has a signficantly reduced burden of suffering on the animals compared to freely-behaving studies where animals have to be fitted with guide cannlulae for a number of days prior to testing. EMG recordings of this type have been used to assess central changes in pain processing previously^25,41^. Rats were anaesthetised with isoflurane (3% in 99% O2) and a tracheotomy performed. Rats were artificially ventilated (Harvard Apparatus, UK) via an endotracheal cannula. Body temperature was maintained at 37 °C using a homeothermic heated blanket (Harvard Apparatus). Rats were mounted on a stereotaxic frame and craniotomies performed over the RVM (anterior-posterior [A-P] −11.0 mm, medial-lateral [M-L] 0.0 mm, dorsal-ventral [D-V] −10.5 mm). A custom-built bipolar concentric EMG electrode was inserted into the belly of the biceps femoris muscle ipsilateral to the MIA/saline injection. The electrode was connected to a NeuroLog head-stage (module NL100AK; Digitimer, Welwyn Garden City, UK), signals amplified x2000 (module NL104A), band-pass filtered between 10–1000 Hz (module NL125) before being sampled at 2 kHz using Spike2 V.7 software via a micro1401 data acquisition unit (CED, Cambridge, UK) as previously described^41^. Isoflurane concentration was reduced stepwise to a final concentration of between 1.0–1.3%, which was determined as being the highest concentration that permitted reproducible pedal reflex responses. Spinally mediated nociceptive paw withdrawal reflexes were evoked by the application of von Frey hairs to the plantar surface of the ipsilateral hindpaw. Mechanical threshold was defined as the minimum mechanical force in grams that evoked an increased EMG response at least 10% above basal activity. Once the threshold mechanical force was determined, von Frey hairs were presented in ascending order of bending force, with two below threshold, one at threshold and one suprathreshold. The magnitude of EMG responses to subthreshold, threshold and supra-threshold stimuli was determined. Each hair was presented 3 times, and 3 evoked baseline responses were measured at 10 minute intervals. EMG recording and pharmacological manipulation of the RVM was performed as previously described^51^. RVM microinjections consisted of administering 200 nl of [D-Ala^2^, N-MePhe^4^, Gly-ol]-enkephalin (DAMGO (Tocris; UK): 3 ng or 30 ng per microinjection) or lidocaine (10 ng, to inactivate neuronal excitability in all cells in the entire NRM) slowly over 5 mins into the RVM, specifically the nucleus raphe magnus. more information see Supplementary Information.

Following both behavioural and EMG studies animals were humanely killed and knees were harvested for histological examination. Animals in the MIA group that did not have verified pathological changes in the injected knee indicative of OA-like processes were removed from all data analyses.

### Macroscopic confirmation of osteoarthritis

Rats were overdosed with sodium pentobarbital (1 ml, intra-periotoneal) and transcardially perfused with saline (0.9%) until exsanguinate ran clear. Infusate Perfusate was then switched to 4% paraformaldehyde (PFA; Sigma, U.K) made up in phosphate buffered saline (PBS). Whole knees (both ipsilateral and contralateral) were dissected and disarticulated and processed in accordance with procedures publihsed previously^18^. Macroscopic scoring of the chondral surfaces was used to confirm the presence of MIA-induced lesions to corroborate behavioural and electrophysiological findings. Macroscopic lesions were graded as follows: 0 = normal appearance; 1 = slight yellowish discoloration of the chondral surface; 2 = little cartilage erosions in load-bearing areas; 3 = large erosions extending down to the subchondral bone; and 4 = large erosions with large areas of subchondral bone exposure [scoring system as used in^52^. Each of the chondral surfaces, medial and lateral femoral condyles, medial and lateral tibial plateaus and the femoral groove, was graded separately by 2 observers. Scores for each of the 5 regions were combined (maximum possible score of 20) and the mean determined for each group.

### Group Sizes and Statistical Analysis

Allocation and blinding: prior to any intervention baseline behavioural measures, WB and PWT, were taken and animals assigned to either MIA/NGF or saline groups so that each group had equivalent means. Within these two groups, rats were assigned into groups for further pharmacological interventions on the basis of their WB and PWT values post the intial intervention so that mean values of these parameters within each group (MIA/NGF or saline) were comparable. Drugs used in this study were prepared by the authors and then anonymised and encoded. The instructions for which coded drug solution was given to each coded animal was then provided to the experimenter. Allocation and blinding was performed by a third party researcher who had no part in the study and is not an author on this publication.

Group sizes for studies were determined using Power Calcualtions using data obtained in our laboratories with the same model of OA (behavioural studies) or using the EMG recording to assess the impact of supraspinal pharmacological manipulations. The minimum group size required for studies with a systemic pharmacological intervention on pain behaviour (mean difference between two groups (drug versus vehicle) was 48.98 (SD 25.05)) was 4 rats per group to detect the difference between intervention and vehicle with 90% power and less than 5% type I error. In these studies experimentors were blind to both intitial treatment (MIA or saline) as well as subsequent intra-articular pharmacological challenge. For EMG recordings the minimum group size for studies comparing reflex magnitude after a supraspinal pharmacological intervention in MIA and saline treated rats (mean difference between groups (baseline versus post-drug) was 49 (SD 36)) with 90% power and less than 5% type I error was 6.

Changes in PWT or WB following MIA or NGF injection were performed using a one-way ANOVA with Dunnett’s multiple comparison test comparing WB and PWT to the baseline (0) measurement. For comparisons between treatment groups after the second intra-articular drug administration (in either the NGF or MIA studies) a 2-way ANOVA (treament x time) was performed with mean responses of each treatment over the entire 180 post-drug period compared between groups with a Dunnett’s multiple comparisons test. Analyses were carried out using Prism 6.0 software (Graphpad, La Jolla, USA). Raw EMG data was extracted using custom-written scripts in MATLAB (R2012a; MathWorks, Natick, MA, USA). Extracted data were imported into Prism and the magnitude and duration of baseline evoked EMG responses was compared between MIA and saline groups at different timepoints. EMG thresholds were compared using a one-way ANOVA with Tukey’s multiple comparison post-test). The effect of each pharmacological intervention on the magnitude of the EMG response was expressed as a percentage of its pre-drug baseline response. Normalised post-drug responses and macroscopic knee scores were compared directly between treatments, interventions and timepoints using two-way ANOVA with Sidak post-tests.

### Data Availability Statement

The datasets generated during and/or analysed during the current study are available from the corresponding author on reasonable request.

## Electronic supplementary material

Supplemental Information

## References

[1] Cross, M. *et al*. The global burden of hip and knee osteoarthritis: estimates from the global burden of disease 2010 study. *Ann Rheum Dis* **73**, 1323–1330, 10.1136/annrheumdis-2013-204763 (2014). 10.1136/annrheumdis-2013-204763 24553908

[2] Javaid MK (2012). Individual magnetic resonance imaging and radiographic features of knee osteoarthritis in subjects with unilateral knee pain: the health, aging, and body composition study. Arthritis Rheum.

[3] Finan PH (2013). Discordance between pain and radiographic severity in knee osteoarthritis: findings from quantitative sensory testing of central sensitization. Arthritis Rheum.

[4] Courtney CA, Lewek MD, Witte PO, Chmell SJ, Hornby TG (2009). Heightened flexor withdrawal responses in subjects with knee osteoarthritis. J Pain.

[5] Suokas AK (2012). Quantitative sensory testing in painful osteoarthritis: a systematic review and meta-analysis. Osteoarthritis Cartilage.

[6] Lluch E, Torres R, Nijs J, Van Oosterwijck J (2014). Evidence for central sensitization in patients with osteoarthritis pain: a systematic literature review. Eur J Pain.

[7] Guermazi A (2014). Synovitis in knee osteoarthritis assessed by contrast-enhanced magnetic resonance imaging (MRI) is associated with radiographic tibiofemoral osteoarthritis and MRI-detected widespread cartilage damage: the MOST study. The Journal of rheumatology.

[8] de Lange-Brokaar BJ (2015). Association of pain in knee osteoarthritis with distinct patterns of synovitis. Arthritis Rheumatol.

[9] Pearle AD (2007). Elevated high-sensitivity C-reactive protein levels are associated with local inflammatory findings in patients with osteoarthritis. Osteoarthritis Cartilage.

[10] Creamer P, Hunt M, Dieppe P (1996). Pain mechanisms in osteoarthritis of the knee: effect of intraarticular anesthetic. The Journal of rheumatology.

[11] Porreca F (2001). Inhibition of neuropathic pain by selective ablation of brainstem medullary cells expressing the mu-opioid receptor. J Neurosci.

[12] Gwilym, S. E., Oag, H. C., Tracey, I. & Carr, A. J. Evidence that central sensitisation is present in patients with shoulder impingement syndrome and influences the outcome after surgery. *The Journal of bone and joint surgery. British volume* **93**, 498–502, doi:10.1302/0301-620×.93B4.25054 (2011). 10.1302/0301-620X.93B4.25054 21464489

[13] Fields HL, Basbaum AI, Clanton CH, Anderson SD (1977). Nucleus raphe magnus inhibition of spinal cord dorsal horn neurons. Brain Res.

[14] Arendt-Nielsen L (2010). Sensitization in patients with painful knee osteoarthritis. Pain.

[15] Graven-Nielsen T, Wodehouse T, Langford RM, Arendt-Nielsen L, Kidd BL (2012). Normalization of widespread hyperesthesia and facilitated spatial summation of deep-tissue pain in knee osteoarthritis patients after knee replacement. Arthritis and rheumatism.

[16] Gregory MH (2012). A review of translational animal models for knee osteoarthritis. Arthritis.

[17] Malfait AM, Little CB (2015). On the predictive utility of animal models of osteoarthritis. Arthritis Res Ther.

[18] Nwosu LN, Mapp PI, Chapman V, Walsh DA (2016). Relationship between structural pathology and pain behaviour in a model of osteoarthritis (OA). Osteoarthritis Cartilage.

[19] Bove S (2003). Weight bearing as a measure of disease progression and efficacy of anti-inflammatory compounds in a model of monosodium iodoacetate-induced osteoarthritis. Osteoarthritis Cartilage.

[20] Sagar DR (2011). The contribution of spinal glial cells to chronic pain behaviour in the monosodium iodoacetate model of osteoarthritic pain. Molecular Pain.

[21] Sagar DR (2010). Tonic modulation of spinal hyperexcitability by the endocannabinoid receptor system in a rat model of osteoarthritis pain. Arthritis Rheum.

[22] Fernihough J (2004). Pain related behaviour in two models of osteoarthritis in the rat knee. Pain.

[23] Kelly S, Dobson KL, Harris J (2013). Spinal nociceptive reflexes are sensitized in the monosodium iodoacetate model of osteoarthritis pain in the rat. Osteoarthritis Cartilage.

[24] Rahman, W. *et al*. Descending serotonergic facilitation and the antinociceptive effects of pregabalin in a rat model of osteoarthritic pain. *Mol Pain* **5**, 45, 10.1186/1744-8069-5-45 (2009). 10.1186/1744-8069-5-45 PMC2744671 19664204

[25] Hathway GJ, Vega-Avelaira D, Fitzgerald M (2012). A critical period in the supraspinal control of pain: opioid-dependent changes in brainstem rostroventral medulla function in preadolescence. Pain.

[26] Woolf CJ (1983). Evidence for a central component of post-injury pain hypersensitivity. Nature.

[27] De Felice M (2011). Engagement of descending inhibition from the rostral ventromedial medulla protects against chronic neuropathic pain. Pain.

[28] Zhang X, Huang J, McNaughton PA (2005). NGF rapidly increases membrane expression of TRPV1 heat-gated ion channels. Embo J.

[29] Binshtok AM, Bean BP, Woolf CJ (2007). Inhibition of nociceptors by TRPV1-mediated entry of impermeant sodium channel blockers. Nature.

[30] Ashraf, S. *et al*. Augmented pain behavioural responses to intra-articular injection of nerve growth factor in two animal models of osteoarthritis. *Ann Rheum Dis*, 10.1136/annrheumdis-2013-203416 (2013). 10.1136/annrheumdis-2013-203416 PMC4145450 23852764

[31] Deising S (2012). NGF-evoked sensitization of muscle fascia nociceptors in humans. Pain.

[32] Kelly S (2012). Spontaneous firing in C-fibers and increased mechanical sensitivity in A-fibers of knee joint-associated mechanoreceptive primary afferent neurones during MIA-induced osteoarthritis in the rat. Osteoarthritis Cartilage.

[33] Kelly, S. *et al*. Increased function of pronociceptive TRPV1 at the level of the joint in a rat model of osteoarthritis pain. *Ann Rheum Dis*, 10.1136/annrheumdis-2013-203413 (2013). 10.1136/annrheumdis-2013-203413 PMC4283626 24152419

[34] Miller, R. E. *et al*. Chemogenetic inhibition of pain neurons in a mouse model of osteoarthritis. *Arthritis Rheumatol*, 10.1002/art.40118. (2017). 10.1002/art.40118 PMC5501168 28380690

[35] Otis C (2017). Spinal neuropeptide modulation, functional assessment and cartilage lesions in a monosodium iodoacetate rat model of osteoarthritis. Neuropeptides.

[36] Saade NE (2012). Brainstem injection of lidocaine releases the descending pain-inhibitory mechanisms in a rat model of mononeuropathy. Exp Neurol.

[37] Burgess SE (2002). Time-dependent descending facilitation from the rostral ventromedial medulla maintains, but does not initiate, neuropathic pain. J.Neurosci..

[38] Mapp PI (2013). Differences in structural and pain phenotypes in the sodium monoiodoacetate and meniscal transection models of osteoarthritis. Osteoarthritis Cartilage.

[39] Okun A (2012). Afferent drive elicits ongoing pain in a model of advanced osteoarthritis. Pain.

[40] Hendiani, J. A. *et al*. Mechanical sensation and pain thresholds in patients with chronic arthropathies. *J Pain* **4**, 203–211, https://doi.org/S1526590003005571 [pii] (2003). 10.1016/s1526-5900(03)00557-1 14622705

[41] Hathway G (2006). A postnatal switch in GABAergic control of spinal cutaneous reflexes. The European Journal of Neuroscience.

[42] Koch SC, Fitzgerald M, Hathway GJ (2008). Midazolam potentiates nociceptive behavior, sensitizes cutaneous reflexes, and is devoid of sedative action in neonatal rats. Anesthesiology.

[43] Heinricher MM, Tavares I, Leith JL, Lumb BM (2009). Descending control of nociception: Specificity, recruitment and plasticity. Brain Res Rev.

[44] Heinricher MM, Morgan MM, Fields HL (1992). Direct and indirect actions of morphine on medullary neurons that modulate nociception. Neuroscience.

[45] Kulkarni B (2007). Arthritic pain is processed in brain areas concerned with emotions and fear. Arthritis Rheum.

[46] Gwilym SE, Filippini N, Douaud G, Carr AJ, Tracey I (2010). Thalamic atrophy associated with painful osteoarthritis of the hip is reversible after arthroplasty: a longitudinal voxel-based morphometric study. Arthritis Rheum.

[47] Rodriguez-Raecke R, Niemeier A, Ihle K, Ruether W, May A (2009). Brain gray matter decrease in chronic pain is the consequence and not the cause of pain. J Neurosci.

[48] Inglis JJ (2008). Regulation of pain sensitivity in experimental osteoarthritis by the endogenous peripheral opioid system. Arthritis Rheum.

[49] Kosek E, Ordeberg G (2000). Lack of pressure pain modulation by heterotopic noxious conditioning stimulation in patients with painful osteoarthritis before, but not following, surgical pain relief. Pain.

[50] Gwilym SE (2009). Psychophysical and functional imaging evidence supporting the presence of central sensitization in a cohort of osteoarthritis patients. Arthritis Rheum.

[51] Hathway GJ, Koch S, Low L, Fitzgerald M (2009). The changing balance of brainstem-spinal cord modulation of pain processing over the first weeks of rat postnatal life. J Physiol.

[52] Guingamp, C. *et al*. Mono-iodoacetate-induced experimental osteoarthritis: a dose-response study of loss of mobility, morphology, and biochemistry. *Arthritis Rheum* **40**, 1670–1679, 10.1002/1529-0131(199709)40:9&lt;1670::AID-ART17&gt;3.0.CO;2-W (1997). 10.1002/art.1780400917 9324022

