# Supplementary material for: Differential contributions of peripheral and central mechanisms to pain in a rodent model of osteoarthritis
Source: Sci Rep. 2018 May 8;8:7122. doi: 10.1038/s41598-018-25581-8 (PMC5940779; doi:10.1038/s41598-018-25581-8)
Supplement: Supplementary file 1 — Supplemental Information [file 41598_2018_25581_MOESM1_ESM.pdf]

Supplemental Information

Differential contributions of peripheral and central mechanisms to pain in a rodent model of osteoarthritis

Adrian R. Haywood PhD, Gareth J. Hathway PhD and Victoria Chapman PhD

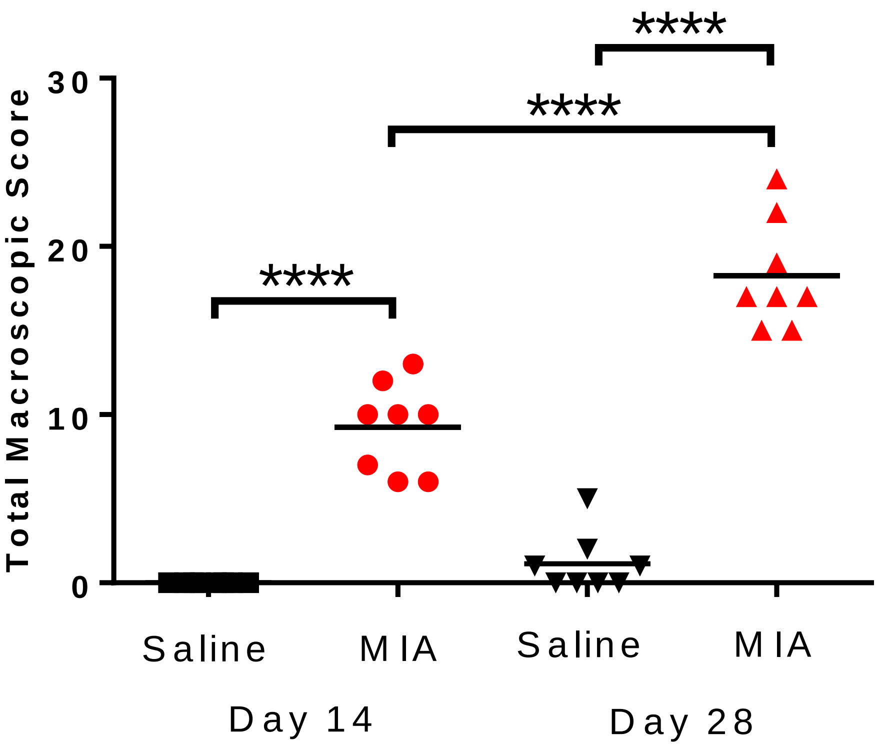

Supplementary Figure 1: Macroscopic scoring was performed on six knee compartments in MIA and saline-treated rats. At Day 14 post-MIA, there was a significant increase in macroscopic score compared to saline-treated rats. At Day 28 MIA treated rats also had a significantly increased score compared to saline controls and compared to Day 14 MIA rats. Treatments were compared with a one-way ANOVA with Sidak post-hoc test. N=8 rats per group ****=p<0.0001.

A

Day-14: Injection sites

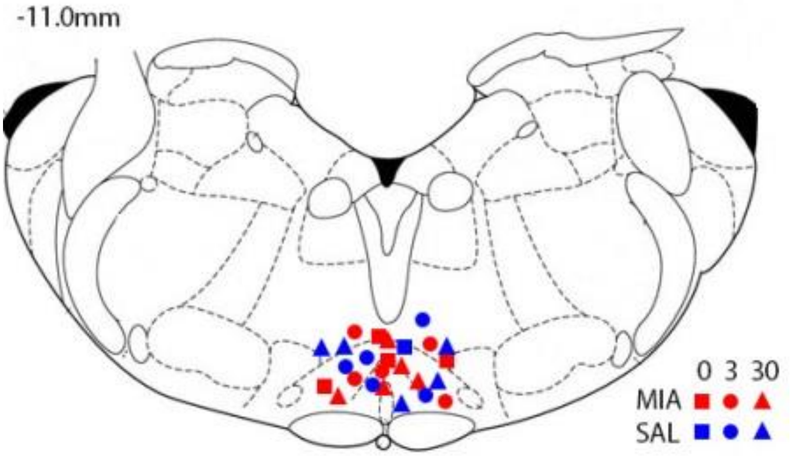

B

Day-28: Injection sites

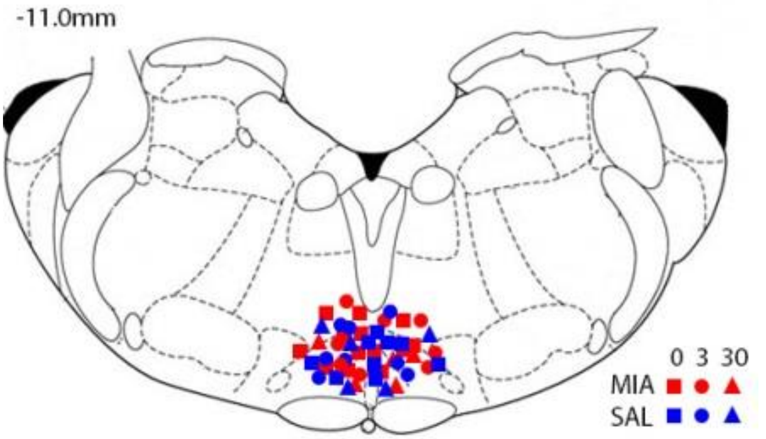

Supplementary Figure 2: Locations of intra-RVM microinjections at both Day 14 and Day 28 in MIA and saline treated rats.

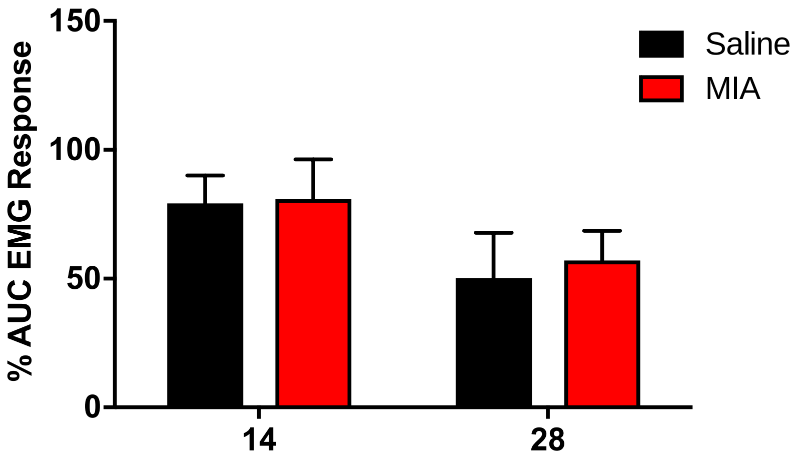

Supplementary Figure 3: Intra-RVM microinjections of lidocaine were unable to alter spinal reflex excitability at either Day14 or 28 post MIA.
